# Gastrodin inhibits neuronal ferroptosis by regulating mir-455–5p/ACSL4 pathway after stroke via exosome

**DOI:** 10.3389/fphar.2026.1882367

**Published:** 2026-10-01

**Authors:** Yinan Nan, Wenhao Zhu, Shaoqing Wang

**Affiliations:** 1 China-Japan Friendship Hospital, Beijing, China; 2 ZiBo Hospital of Traditional Chinese Medicine, Zibo, China; 3 Beijing Tiantan Hospital Affiliated to Capital Medical University, Beijing, China

**Keywords:** ACSL4, exosome, ferroptosis, GPx4, ischemic stroke, miR-455–5p

## Abstract

Recent studies have demonstrated that ferroptosis plays a crucial role in neuronal cell death following cerebral infarction. Our previous research showed that gastrodin (GAS) significantly improved neurological function in rats subjected to middle cerebral artery occlusion (MCAO); however, the underlying mechanisms remain unclear. In the present study, we employed Western blotting, quantitative PCR (qPCR), and immunofluorescence assays to investigate the changes in the expression levels of ferroptosis-related regulatory proteins (ACSL4, TFR1, GPX4, and MBL-2) in both *in vivo* and *in vitro* models following ischemic-hypoxic injury, as well as the modulatory effects of gastrodin on these proteins. We also conducted an in-depth exploration of an innovative therapeutic approach involving exosomes. Specifically, we constructed gastrodin-treated serum exosomes (GSE) to assess their role in neuroprotection. Our findings revealed that gastrodin significantly reduced infarct volume in middle cerebral artery occlusion rats. Compared with the model group, the levels of ACSL4, TFR1, and MBL-2 proteins were markedly decreased, while GPX4 expression was significantly upregulated in the ischemic hemisphere of the gastrodin-treated rats. These results suggest that gastrodin exerts neuroprotective effects by inhibiting ferroptosis following cerebral infarction. In an oxygen-glucose deprivation model using Neuro-2a cells, we further observed that GSE enhanced cell viability by suppressing ferroptosis. Using RNA immunoprecipitation dual-luciferase reporter assays, and fluorescence *in situ* hybridization we confirmed colocalization between miR-455–5p and ACSL4. Subsequently, we manipulated miR-455–5p expression in Neuro-2a cells through transfection and assessed cell viability using CCK-8 and Live/Dead assays. The results demonstrated that GSE inhibited OGD-induced ferroptosis and improved cell viability by modulating the miR-455–5p/ACSL4 signaling pathway. In conclusion, our study indicates that gastrodin can suppress neuronal ferroptosis probably through an exosome-mediated mechanism involving the miR-455–5p/ACSL4 pathway, thereby exerting neuroprotective effects after ischemic injury. In addition, exosomal delivery represents a promising strategy to treat stroke.

## 1 Introduction

Ischemic stroke remains a leading cause of death and long-term disability worldwide (GBD 2019 Stroke Collaborators, 2021), characterized by the sudden loss of blood supply to the brain, leading to extensive neuronal injury and neurological dysfunction (Campbell and Khatri, 2020; Campbell et al., 2019). Despite advances in reperfusion therapies, such as thrombolysis and thrombectomy, the therapeutic window is narrow, and the efficacy remains limited for many patients (Powers et al., 2019). Therefore, exploring novel neuroprotective mechanisms and treatment strategies remains a major priority in stroke research.

Among the emerging mechanisms of neuronal death, ferroptosis has gained increasing attention in recent years. Ferroptosis is a distinct, iron-dependent form of regulated cell death characterized by excessive lipid peroxidation and glutathione depletion (Dixon et al., 2012). It has been implicated in ischemia-reperfusion injury, neurodegeneration, and other oxidative stress–related pathologies (Jiang et al., 2021). In ischemic stroke, iron accumulation and redox imbalance amplify lipid peroxidation, leading to ferroptotic death of neurons and glial cells (Tuo et al., 2017). Inhibition of ferroptosis has been shown to mitigate neuronal loss and improve outcomes in experimental stroke models, suggesting it as a promising therapeutic target (Alim et al., 2019).

Acyl-CoA synthetase long-chain family member 4 (ACSL4) is a critical metabolic enzyme that dictates cellular sensitivity to ferroptosis. ACSL4 acts as an indispensable pro-ferroptotic regulator by synthesizing the lipid substrates required for this process (Doll et al., 2017). ACSL4 preferentially catalyzes the esterification of long-chain polyunsaturated fatty acids (PUFAs)-most notably arachidonic acid (AA) and adrenic acid (AdA)-into their respective acyl-coenzyme A (CoA) derivatives, such as arachidonoyl-CoA (Kagan et al., 2017). This enzymatic conversion is the rate-limiting step in the remodeling of cellular membranes. Following ACSL4 activation, lysophosphatidylcholine acyltransferase 3 (LPCAT3) incorporates these PUFA-CoAs into membrane phospholipids, primarily forming phosphatidylethanolamine-PUFA (PE-PUFA) (Dixon et al., 2015).

Glutathione peroxidase 4 (GPX4) is a pivotal antioxidant enzyme that detoxifies lipid hydroperoxides, thereby preventing ferroptosis (Yang et al., 2014). Reduced GPX4 activity or glutathione (GSH) depletion sensitizes neurons to ferroptotic injury, while GPX4 activation confers protection. Modulating GPX4 expression and activity has thus become a central strategy in ferroptosis-targeted neuroprotection (Conrad and Friedmann Angeli, 2015).

Gastrodin (GAS), a bioactive phenolic glycoside derived from Gastrodia elata Blume, has been traditionally used in Chinese medicine for neurological disorders such as stroke, epilepsy, and cognitive impairment. Modern pharmacological studies have revealed that gastrodin exerts antioxidant, anti-inflammatory, and anti-apoptotic effects in the central nervous system (Shi et al., 2024). Recent evidence suggests that gastrodin may modulate ferroptosis-related pathways, particularly those involving xCT/GPX4 and ACSL4/LPCAT3 signaling (Gong et al., 2025). However, the precise molecular mechanisms by which gastrodin influences ferroptosis during ischemic stroke remain poorly understood.

In recent study, microRNAs (miRNAs) transported by exosomes play crucial roles in regulating neuronal ferroptosis and oxidative stress (Qin et al., 2024). In particular, miR-455–5p has been implicated in protecting neurons from oxidative injury by reducing the neuroinflammatory response (Zhang et al., 2022). However, whether gastrodin can regulate ferroptosis through the exosome-mediated pathway has not been elucidated.

Therefore, in this study, we investigated the neuroprotective mechanism of gastrodin in cerebral ischemia, and the role gastrodin-treated serum exosime plays in it. We aimed to determine whether GSE could suppress ferroptosis and enhance neuronal survival by modulating the miR-455–5p/ACSL4 signaling pathway. This study not only provides new mechanistic insights into the anti-ferroptotic effects of gastrodin but also highlights the potential of exosome-mediated delivery systems for treating ischemic stroke.

## 2 Materials and methods

### 2.1 Mice and focal cerebral ischemia

SD rats (body weight, 280–300 g) were purchased from Vital River Laboratory Animal Technology Co. Ltd., Beijing, China. All animal procedures were performed according to the China Animal Welfare Legislation and approved by the Capital Medical University Committee on the Care and Use of Laboratory Animals (approval number: AEEI-2023–173). Rats were anesthetized with pentobarbital (50 mg/kg, intravenous injection), and then fixed in a supine position on the operating table. The fur was removed from the surgical site, and the area was disinfected with iodine. A midline cervical incision was made to expose the left common carotid artery (CCA), the left external carotid artery (ECA), and the internal carotid artery (ICA). The proximal end of the CCA and the distal end of the ECA were ligated. A suture was inserted into the ICA until slight resistance was encountered, at which point the procedure was stopped. After successful modeling of middle cerebral artery occlusion (MCAO), the timing for reperfusion was set at 60 min. The procedure was unilateral.

Successful induction of ischemia was evaluated 60 min after reperfusion using the modified Longa five-point neurological scoring system. Rats with scores of 0 (no deficit) or 4 (unable to walk spontaneously) were considered unsuccessful modelings or severe subarachnoid hemorrhage, respectively, and were excluded from the study. All excluded animals were replaced to ensure an adequate sample size.

The model group received daily intraperitoneal injections of saline, while the gastrodin group received daily intraperitoneal injections of gastrodin (100 mg/kg). At the conclusion of the experimental period, the rats were humanely euthanized via an intraperitoneal overdose of pentobarbital (150 mg/kg) prior to the collection of brain tissues.

### 2.2 Drug treatment

Gastrodin was purchased from the Shanghai Tongtian Pharmaceutical Corporation. GAS was dissolved in saline and administered intraperitoneally once a day for 7 days. GAS dose was 100 mg/kg 7 days later, serum was collected to prepare GSE for subsequent animal and cellular experiments.

### 2.3 Antibodies and reagents

The following antibodies from Affinity Biosciences were used: ACSL4 (DF12141), TFR1 (AF5343), GPX4 (DF6701), MBL2 (DF4152), β-actin (AF7018).

### 2.4 Primary cell culture and OGD treatment

Neuro-2a cells were obtained from the American Type Culture Collection (ATCC, Cat# CCL-131, Manassas, VA, USA). Cells were cultured in Eagle’s Minimum Essential Medium (EMEM) supplemented with 10% fetal bovine serum (FBS, Gibco) and 1% penicillin-streptomycin at 37 °C in a humidified atmosphere containing 5% CO_2_. All experiments were performed using cells at passages ≤15. Routine *Mycoplasma* testing was conducted every 2 weeks using a *Mycoplasma* Detection Kit (Lonza, Cat# LT07-318) based on PCR amplification, and all tested samples were confirmed to be negative prior to use. The OGD challenge was performed using previously established protocols (Liu et al., 2010). Cells were transferred to deoxygenated glucose-free extracellular solutions (in mM: 116 NaCl, 5.4 KCl, 0.8 MgSO4, 1.0 NaH2PO4, 1.8 CaCl2 and 26 NaHCO3); introduced into a dedicated chamber (Plas-Labs, Lansing, USA) and held in 95% N2/5% CO2 at 37 °C for 90 min. The medium was then replaced with fresh maintenance medium containing the appropriate concentration of reagents for the indicated times during recovery in a 95% O2/5% CO2 incubator. Control cultures were first transferred to another extracellular solution (in mM/L: 116 NaCl, 5.4 KCl, 0.8 MgSO4, 1.0 NaH2PO4, 1.8 CaCl2, 26 NaHCO3, and 33 glucose), and humidified at 37 °C for 90 min in 95% O2/5% CO2. The entire medium was then replaced with fresh maintenance medium in a 95% O2/5% CO2 incubator at 37 °C. Following the hypoxic period, the medium was replaced with fresh normoxic maintenance medium containing the indicated concentrations of GSE (or controls), and the cells were returned to a normoxic incubator (95% O_2_/5% CO_2_) for reoxygenation. Unless otherwise specified, all subsequent assays were performed at 24 h post-reoxygenation.

### 2.5 CCK8 assay

To evaluate cellular survival, a Cell Counting Kit-8 (Solarbio) assay was utilized in accordance with the manufacturer’s protocol. Briefly, Neuro-2a cells were seeded into 96-well culture plates. Following the designated experimental treatments, the CCK-8 reagent was introduced to each well, and the plates were subsequently incubated for a period of 1–4 h. Cellular viability was quantified by assessing the optical density (OD) at a wavelength of 450 nm, and the final results were expressed as a percentage relative to the untreated control group.

### 2.6 Immunoblot analysis

Cells were lysed and incubated at 100 °C for 15 min. Proteins were separated by SDS-PAGE and transferred to nitrocellulose membranes (GE Healthcare). Membranes were blocked with 5% milk in Tris buffered saline for 60 min at room temperature. Membranes were incubated with primary and secondary antibodies. Membranes were visualized by immunoblot analysis with the chemiluminescence detection system.

### 2.7 Quantitative real-time PCR (qRT-PCR) analysis

Total RNA of brain tissues was isolated using TRIzol reagent (Ambion) according to the manufacturer’s protocol. Total RNA of cultured cells was extracted with RNAfast200 purification kit (Fastagen), and reverse-transcribed with the ExonScript RT SuperMix with dsDNase (EXONGEN). Real-time PCR was performed on the Biorad 96 touch machine (Biorad) with 2× RealStar Green Power Mixture (GenStar). U6 was used as the internal control. The sequences of qRT-PCR primers for the genes examined are listed below: ACSL4 forward primer:5′-TAAGTACAACTTTCCGCTTGTG-3′; reverse primer: 5′-ATA​GGA​AGC​CTC​AGA​CTC​A-3′; TFR1 forward primer: 5′- TCA​GGT​AGT​AAC​ATT​GAC​CCA-3′; reverse primer: 5′- GTG​CCA​AAA​TTA​GCA​TGG​AC-3′; GPX4 forward primer: 5′- CCC​ATT​CCC​GAG​CCT​TTC​AAC​C -3′; reverse primer: 5′- ATC​GAT​GTC​CTT​GGC​TGC​GAA​T-3′; U6 forward primer: 5′- GGA​ACG​ATA​CAG​AGA​AGA​TTA​GC-3′; reverse primer: 5′-AAA​TAT​GGA​ACG​CTT​CAC​GA -3′.

### 2.8 Immunohistology and quantification

Brain tissues were fixed in 4% PFA in phosphate buffer for 12 h at room temperature (RT) and incubated at 4 °C in phosphate buffer containing 30% sucrose for 48 h. The brains were frozen in optimum cutting temperature compound and sectioned at 40 μm. The cryosections were first permeabilized with 0.3% triton-X-100 for 30 min, blocked in PBS containing 10% goat serum for 2 h and incubated at 4 °C overnight with the primary antibody against ACSL4 (1:200, Santa Cruz Biotechnology), NeuN (1:500, Abcam), GFAP (1:500, DAKO), Iba-1 (1:500, Wako). After several PBS washes, sections were stained with secondary antibodies for 1 h. The nuclei were stained with DAPI (S2110, Solarbio). A TUNEL assay was performed using a one-step Apoptosis Fluorescein Detection Kit (C1086; Beyotime) according to the manufacturer’s protocol. The TUNEL-positive cells were evaluated under microscopy. Slides were taken with a Leica confocal microscope TCS SP8 or Nikon C2 and analyzed using ImageJ software. To quantify colocalization, images were analyzed by using ImageJ Coloc2 and Pearson’s correlation coefficient (r) values were calculated to measure the correlation dependency of colocalized voxels between separate colour channels.

### 2.9 Immunofluorescence staining

To characterize cells according to immunofluorescence, the Neuro-2a cells were briefly washed with PBS, fixed in 4% paraformaldehyde for 15 min, and then washed 3 times with PBS for 5 min each. Neuro-2a cells were treated with 0.3% Triton-100 for 10 min and blocked with 5% BSA for 30 min. Then, Neuro-2a cells were incubated with a rabbit anti-GPX4 primary antibody at 4 °C overnight. After washing with PBS, the Neuro-2a cells were incubated with a secondary antibody for 1 h at room temperature. To visualize nuclei, DAPI staining was performed. Images were captured using a fluorescence microscope.

### 2.10 Exosome preparation and identification

In this study, exosomes were extracted from rat serum using ultracentrifugation and characterized by transmission electron microscopy (TEM), Nanoparticle tracking analysis (NTA), and Western blot. Serum of nine donor rats in each group was collected and mixed, total volume was 18 mL (control group) and 20 mL (gastrodin group). The process began by thawing the serum sample at 37 °C and then transferring it to a new centrifuge tube for centrifugation at 2000 *g*, 4 °C, for 30 min to remove cellular debris. The supernatant was collected and subjected to further centrifugation at 10,000 × g, 4 °C, for 45 min to remove larger vesicles. The supernatant was then filtered, and the filtrate was subjected to ultracentrifugation using ultra centrifuge (Hitachi-CP100MX, 100,000 × g, 4 °C, for 70 min) to isolate the exosome pellet. The pellet was resuspended in pre-chilled 1×PBS and subjected to another round of ultracentrifugation to further purify the exosomes. The final exosome sample was resuspended in 500 μL PBS, aliquoted at 50 μL per tube, and a portion was used for TEM imaging, NTA and Western blot. All samples were stored at −80 °C. Samples underwent one freeze-thaw cycle prior to characterization and functional experiments. For TEM analysis, the extracellular vesicles (EVs) sample was deposited on a copper grid, stained with uranyl acetate, and imaged. This method ensured the extraction and reliable characterization of high-purity exosomes from rat serum.

### 2.11 Transmission electron microscopy (TEM)

Take 10 μL of the EVs sample, drop it onto the copper slide and let it settle for 1 min. Then, use a filter paper to absorb the supernatant, add 10 μL of acetic diuranate, and again use the filter paper to absorb the supernatant. Let it dry at room temperature for several minutes, and then the samples were observed with the HT7800 transmission electron microscope (Hitachi). Randomly chosen fields for each sample were observed.

### 2.12 Malondialdehyde (MDA) assay

To quantitatively assess the extent of lipid peroxidation, malondialdehyde (MDA) levels were measured in both brain tissue homogenates and Neuro-2a cell lysates using a Lipid Peroxidation (MDA) Assay Kit (Nanjing Jiancheng Bioengineering Institute, A003-4–1) based on the instruction of manufacturer. All measurements were performed in triplicate.

### 2.13 Nanoparticle tracking analysis (NTA)

Nanoparticle tracking analysis (NTA) was performed using a ZetaView PMX120 instrument (Particle Metrix, Germany) to determine the particle size distribution and concentration of the isolated exosome samples. The instrument was warmed up for 30 min, and the detection cell was rinsed with ultrapure water to minimize background particle interference. The sample was then introduced, and measurements were acquired using a 488 nm laser in light-scattering mode. By recording the Brownian motion trajectories of individual particles, the NTA software calculated the particle size and concentration based on the Nanoparticle Tracking Analysis technology.

### 2.14 R-IP

In this study, RNA immunoprecipitation (R-IP) was employed to investigate the interaction between miR-455–5p and ACSL4 in the model group and the Gastrodin-treated group of rats. First, at least 100 mg of tissue samples were collected from each group and lysed with an equal volume of RIP lysis buffer, containing protease inhibitors and RNase inhibitors. The samples were mixed thoroughly and incubated on ice for 10 min. After cell lysis, the lysate was incubated with magnetic beads, and positive control (Input group), target protein antibody group, and negative control (IgG group) were set up. Each group of magnetic beads was washed twice with RIP wash buffer to remove impurities. The beads were then incubated with the target antibody (5 μg) on a shaker for 30 min, followed by a second wash with RIP wash buffer and stored at 4 °C. The tissue lysate was stored at −80 °C, rapidly thawed, and centrifuged at 13,000 rpm for 10 min. The supernatant was collected, and 10 μL was reserved as the Input group for later analysis. The remaining supernatant was mixed with RIP immunoprecipitation buffer (a mixture of RIP wash buffer, 0.5 M EDTA, and RNase inhibitor) and incubated at 4 °C overnight. Magnetic beads were separated using a magnetic stand, and the supernatant was discarded. The beads were washed six times with RIP wash buffer. Subsequently, proteinase K buffer was added, and the mixture was incubated at 55 °C for 30 min. Afterward, chloroform and ethanol were used to extract RNA, which was then subjected to reverse transcription. The extracted RNA was quantified using qPCR to assess the abundance of the target RNA. This method effectively validated the interaction between miR-455–5p and ACSL4, providing substantial experimental data for subsequent analyses.

### 2.15 Fluorescence *in situ* hybridization

The fluorescence *in situ* hybridization (FISH) assay was performed to observe the intracellular localization of the miRNAs. The Neuro-2a cells in Model group and Gastrodin group were isolated and frozen and embed in the Tissue-Tek to cut into 10 μM cryostat sections. After fixed and acetylated, the sections were washed and subject to *in situ* hybridization. FAM-labeled miR-455–5p and Cy3-labeled ACSL4 probes were used in the hybridization. The signals were detected using a tyramide signal amplification (TSA) kit (Gefan, Shanghai, China). Nuclei were counterstained with DAPI. Images were acquired on a Nikon ELCIPSE-CI confocal microscope (Nikon, Japan).

### 2.16 *In vivo* fluorescence imaging system

In this study, exosomes were labeled with the near-infrared lipophilic dye DiR (40757 ES, Yeasen) for *in vivo* fluorescence imaging and tracking in rat. The detailed procedure was as follows: purified exosomes were mixed with DiR working solution and incubated at 37 °C in the dark for 30 min. After incubation, free unbound dye was removed by ultracentrifugation at 120,000 × g for 1 h. The labeled exosomes were resuspended in PBS and injected into rats via the tail vein. *In vivo* imaging was performed using an IVIS small animal imaging system (invivosmart-LF, VLEWORKS) with an excitation/emission filter set of 710 nm/760 nm and an exposure time of approximately 10 s. The whole-body biodistribution of the exosomes was dynamically monitored at multiple predetermined time points post-injection.

### 2.17 Statistical analysis

The statistical analysis was performed using GraphPad Prism soft-ware. All experiments were performed three or more times unless otherwise indicated. All data were shown as Means ± S.D. To compare the statistical significance of two independent groups, Student’s t-test was used. Differences between the groups were determined using ANOVA with Bonferroni *post hoc* test.

All experiments were performed as three independent biological replicates (n = 3), unless otherwise stated. Each biological replicate represents an independent preparation of small extracellular vesicles from a separate group of donor rats (for *in vivo* experiments) or an independent passage of Neuro-2a cells (for *in vitro* experiments). Technical replicates were performed within each biological replicate to ensure assay precision; the average of technical replicates was used as a single data point for statistical analysis.

All data are expressed as mean ± standard deviation (SD). Statistical analyses were performed using GraphPad Prism software. Normality of data distribution was assessed using the Shapiro–Wilk test, and homogeneity of variances was assessed using Levene’s test. For comparisons between two independent groups, unpaired two-tailed Student’s t-test was applied. For comparisons among three or more groups, one-way analysis of variance (ANOVA) was used, followed by Bonferroni’s *post hoc* correction for multiple comparisons when the overall ANOVA indicated a significant difference. A P value of <0.05 was considered statistically significant.

## 3 Results

### 3.1 Gastrodin can reduce infarction volume of MCAO rats

Our previous research has confirmed that gastrodin can improve the neurological function of MCAO rats (Nan et al., 2023). To further verify the conclusion, we employed animal MRI method to detect the infarction area in rats. We measured infarct volume 1 day after surgery, followed by 7 days drug administration, then we measured again. The results showed that the infarction volume of gastrodin group was significantly lower than that of model group (Figure 1A).

**FIGURE 1:**
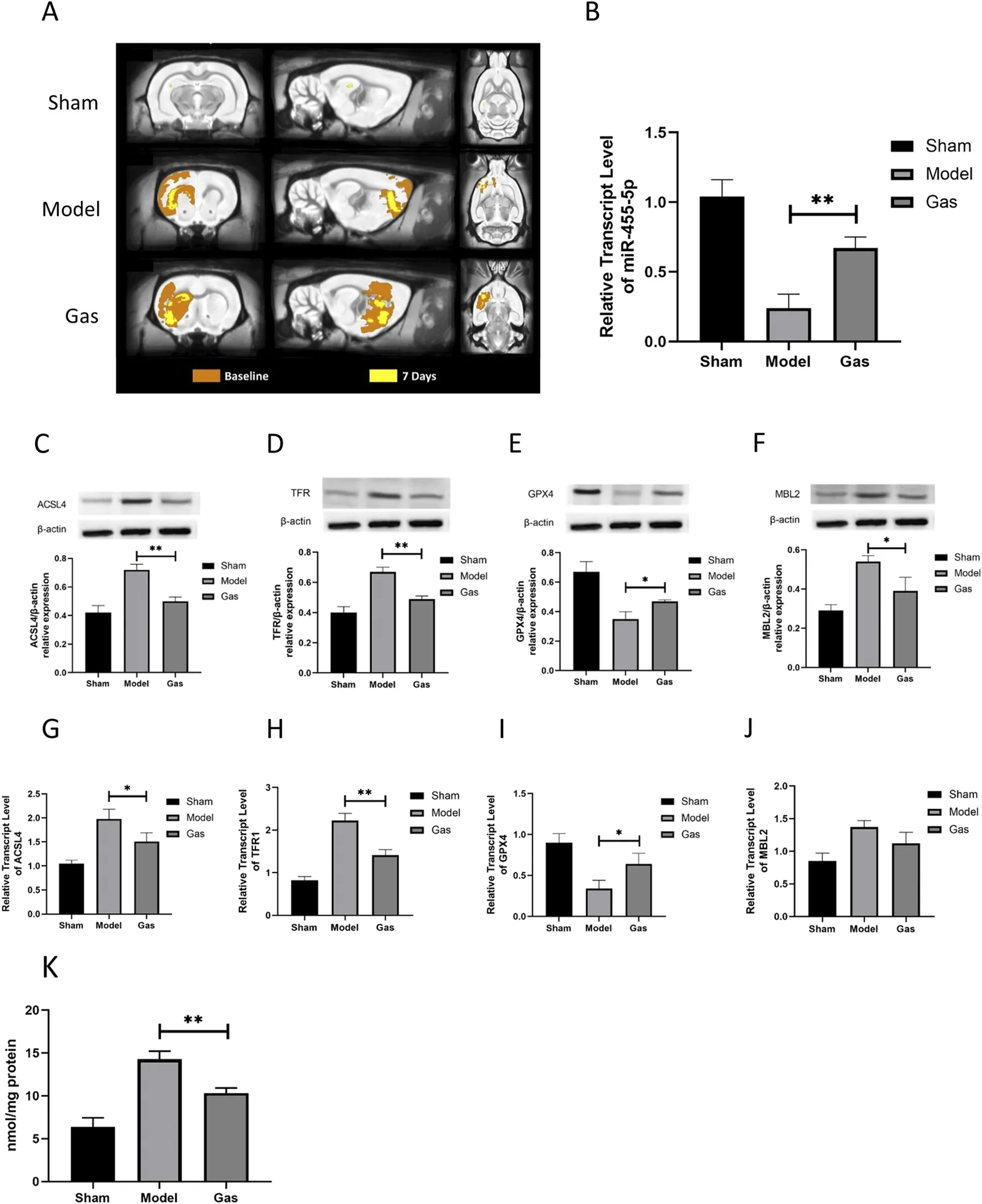
Gastrodin can inhibit ferroptosis in the ischemic hemisphere of MCAO rats. **(A)** MRI results showed infarction volume of gastrodin group was lower than model group in MCAO rats in 7 days (*n* = 3). **(B)** Transcription level of miR-455–5p in gastrodin group was much higher than that in model group. **(C–F)**Representative immunoblot and quantification of ACSL, TFR1, GPX4, MBL2 in ischemic hemisphere of MCAO rats. β-actin band was the same for comparison. **(G–J)** Transcription level of ACSL, TFR1, GPX4, MBL2 in ischemic hemisphere of MCAO rats. **(K)** MDA assay showed gastrodin treatment significantly reduced the MDA level compared with the model group (*n* = 3). *P < 0.05, **P < 0.01.

### 3.2 Gastrodin can inhibit ferroptosis in the ischemic hemisphere of MCAO rats

To investigate the mechanism in which gastrodin can reduce the volume of cerebral infarction, we established tMCAO rat model, rats in gastrodin group were administrated gastrodin (100 mg/kg) intraperitoneally every day, while rats in model group were administrated equal volume of normal saline. After 7 days, rats were sacrificed and ischemic hemispheres were collected. Pivotal ferroptosis regulating proteins were detected by various methods. The results of Western blot showed that protein level of ACSL4, TFR1 and MBL2 in model group were higher than sham group, and that in gastrodin group was significantly lower than model group (Figures 1C,D,F). Protein level of GPX4 in model group were lower than sham group, and that in gastrodin group was significantly higher than model group (Figure 1E). In addition, results of qPCR were consistent with that, it revealed the same trend as protein (Figures 1G–J). Results of MDA assay also indicated gastrodin could inhibit ferroptosis in MCAO rats (Figure 1K). We also detected the amount of miR-455–5p in ischemic hemispheres, the result showed amount of miR-455–5p in model group was much lower than that in sham group, gastrodin can increase the transcription level significantly (Figure 1B).

### 3.3 Gastrodin-treated serum exosome (GSE) can increase cell viability of Neuro-2a cells after oxygen-glucose deprivation (OGD) by inhibiting ferroptosis

We extracted exosome by means of supercentrifugation as mentioned above, obtaining GSE and NE. Then we carried out the identification using transmission electron microscopy methods, nanoparticle tracking analysis (NTA) and Western blot of Calnexin, CD63 and ApoA1 (Supplementary Figure S1A-C).

We used qPCR to detect the amount of miR-455–5p in both GSE and NE, the result revealed much higher miR-455–5p level in GSE than that in NE (Figure 2A). OGD model was established in Neuro-2a cell to evaluate cell viability and the effect of GSE, CCK-8 assay showed cell viability decreased sharply after subjected to OGD in Neuro-2a cells, medium dosage (50 μg/ml) and high dosage (100 μg/ml) GSE can rescue cells markedly, while NE exerted no effect (Figure 3A).

**FIGURE 2:**
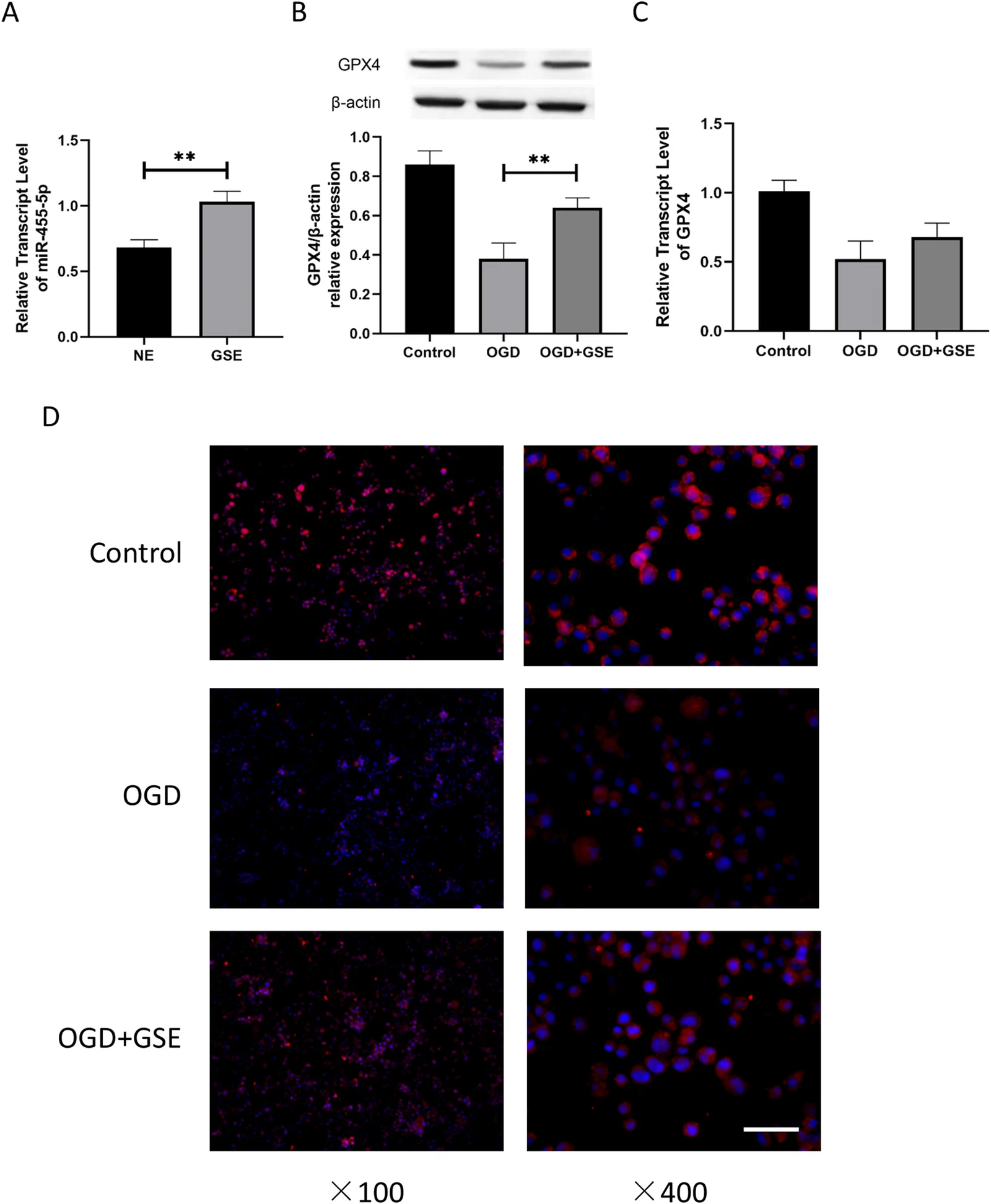
Gastrodin-treated Serum Exosome (GSE) can increase level of GPX in Neuro-2a after OGD. **(A)** Amount of miR-455-5p in GSE was higher than that in NE. **(B)** Representative immunoblot and quantification of GPX4 in Neuro-2a. **(C)** Transcription level of GPX4 in Neuro-2a. **(D)** Representative confocal images of immunofluorescence of GPX4 in Neuro-2a. (n = 3). *P < 0.05, **P < 0.01. Scale bar 20μm.

**FIGURE 3:**
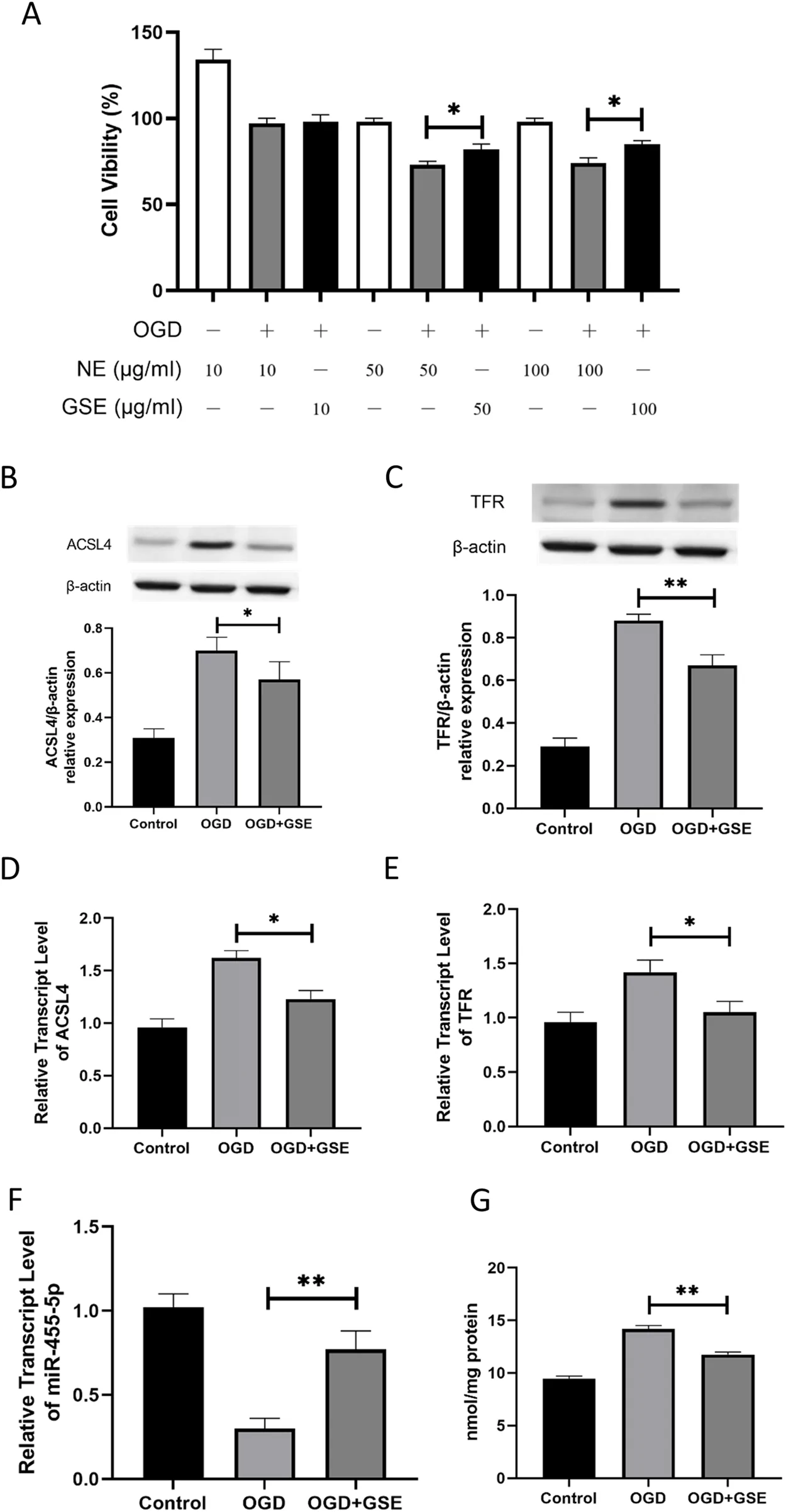
Gastrodin-treated Serum Exosome (GSE) can increase cell viability of Neuro-2a cells after oxygen-glucose deprivation (OGD) by inhibiting ferroptosis. **(A)** CCK-8 assay showed cell viability decreased sharply after subjected to OGD in Neuro-2a cells, medium dosage (50 μg/ml) and high dosage (100 μg/ml) GSE can rescue cells. **(B,C)** Representative immunoblot and quantification of ACSL4 and TFR1 in Neuro-2a. β-actin band was the same for comparison. **(D–F)** Transcription level of ACSL4, TFR1 and miR-455–5p in Neuro-2a after subjecting to OGD. **(G)** MDA assay showed GSE significantly reduced the MDA level compared with the OGD group in Neuro-2a cells. N = 3. *P < 0.05, **P < 0.01.

Then we explored the effect of GSE on ferroptosis regulating protein in Neuro-2a cell. MDA assay showed GSE could inhibit ferroptosis after OGD (Figure 3G). Western blot showed expression level of ACSL4 and TFR1 elevated in Neuro-2a cell after OGD treatment, however, they both decreased after adding medium dose of GSE (Figures 3B,C). As for GPX4, the protein level decreased after OGD treatment, GSE can restore protein level of GPX4 in Neuro-2a cell (Figure 2B). The result of qPCR was in consistent with it (Figure 2C). We also detected the amount of miR-455–5p in Neuro-2a cell, the result showed amount of miR-455–5p in OGD group was much lower than that in control group, GSE can increase the transcription level significantly (Figure 3F).

Result of immunofluorescence showed the expression of GPX4 in OGD group was lower than control group, and GPX4 expression in GSE group was significantly higher compared with OGD grpup (Figure 2D).

To further explore the role ferroptosis plays in GSE increasing cell viability of Neuro-2a after OGD treatment, we introduced ferroptosis inhibitor (Lip-1) and ferroptosis inducer (Erastin) to conduct the experiment. As shown in Figure 4A, compared with the control group, after the addition of the ferroptosis inducer Erastin, the cell activity decreased. In the meantime, cells were subjected to OGD stress followed by Erastin treatment, result of CCK-8 assay revealed that cell viability dropped drastically, notably, it can be rescued by GSE (Figure 4A). Then we verified the result using live/dead assay, it showed the same trend as CCK-8 assay (Figure 4B), GSE can rescue Neuro-2a cell after OGD stress by inhibiting ferroptosis.

**FIGURE 4:**
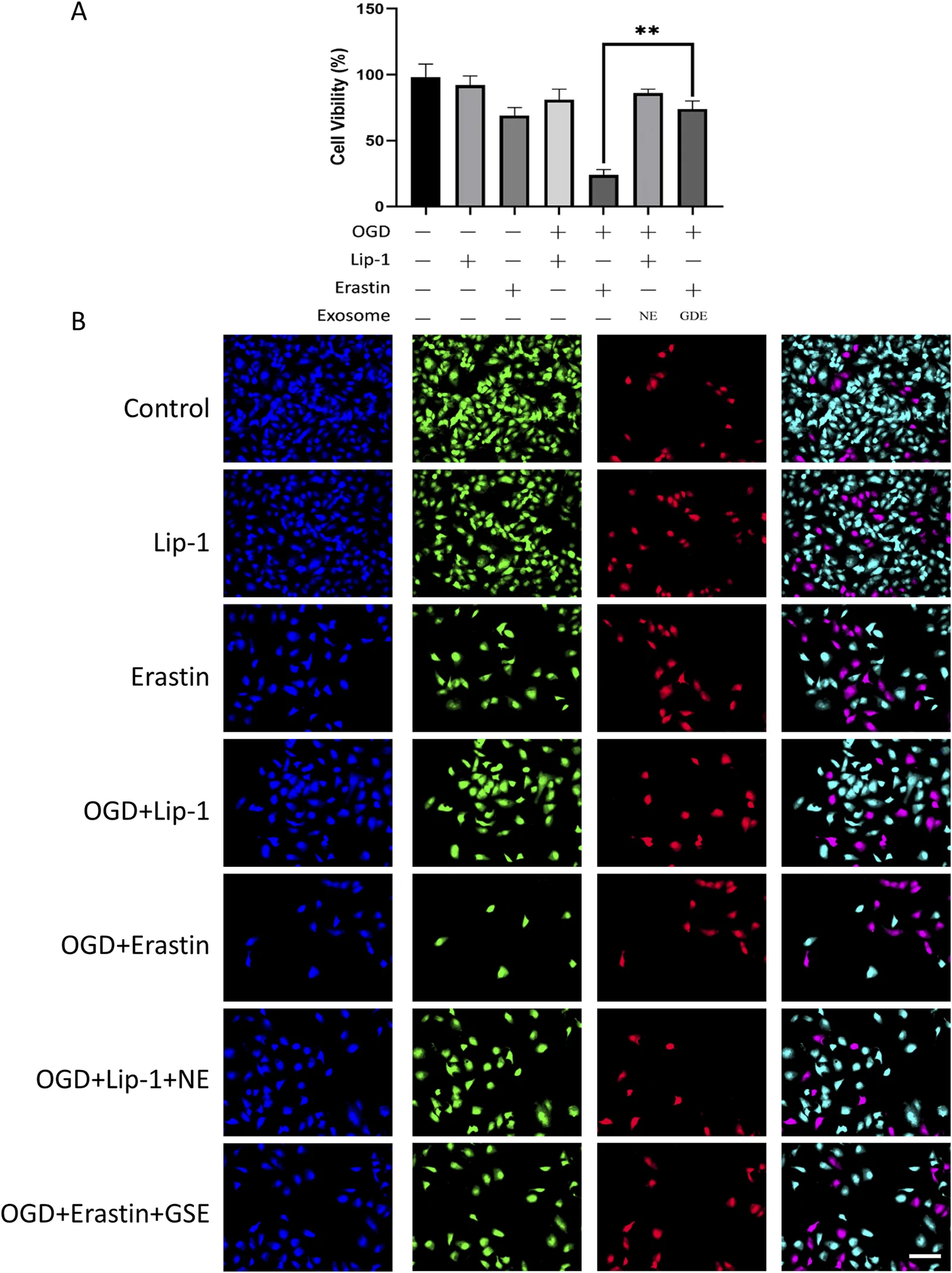
Gastrodin-treated Serum Exosome (GSE) can rescue Neuro-2a cells after oxygen-glucose deprivation (OGD) by inhibiting ferroptosis. **(A)** CCK-8 assay showed cell viability changed after subjecting to ferroptosis inhibitor (Lip-1) and ferroptosis inducer (Erastin). **(B)** Result of live/dead assay showed GSE can rescue Neuro-2a after inducing ferroptosis (n = 3). *P < 0.05, **P < 0.01. Scale bar 20 μm.

The blood-brain barrier (BBB) is a highly selective, protective interface that separates the circulating blood from the brain’s extracellular fluid. It is formed primarily by tightly connected endothelial cells lining the brain’s capillaries, along with supporting cells such as pericytes, astrocytic end-feet, and a basement membrane. It is well known that gastrodin cannot pass through the blood-brain barrier. So how gastrodin takes effect after stroke need to be further studied. Our hypothesis is gastrodin inhibits ferroptosis in the infarcted cerebral hemisphere via exosome. We used the method of *in vivo* imaging of animals to prove that DIR-labeled EV-associated signals were detected in the ischemic brain hemispheres of MCAO rats after 24 h (Supplementary Figure S2). Both NE and GSE can be detected in ischemic hemisphere. CD63 is exosome-specific marker protein, we also detected the amount of CD63 in ischemic hemisphere of MCAO rat by various methods (Western blot, qPCR, immunofluorescence), the results shed light on how gastrodin take effect on MCAO rat (Supplementary Figure S3).

### 3.4 Gastrodin-treated serum exosome (GSE) can inhibit ferroptosis by regulating miR-455–5p/ACSL4 pathway via exosome

Based on the dual-luciferase reporter assay, we first investigated whether miR-455–5p could directly regulate ACSL4 expression through binding to the ACSL4 mRNA 3′-UTR. The results showed that co-transfection of psiCHECK2-ACSL4-WT with miR-455–5p mimic significantly reduced luciferase activity, indicating that miR-455–5p directly targets the 3′-UTR of ACSL4 mRNA.

To further determine whether miR-455–5p was associated with ACSL4 protein complexes, RNA immunoprecipitation (RIP) assay was performed in MCAO rats. The qPCR results showed that the level of miR-455–5p in the immunoprecipitated samples from both the model and gastrodin groups was lower than that in the Input samples, indicating that miR-455–5p was not significantly enriched in ACSL4-associated complexes. These results suggested that miR-455–5p does not directly interact with ACSL4 protein (Figures 5A,B).

**FIGURE 5:**
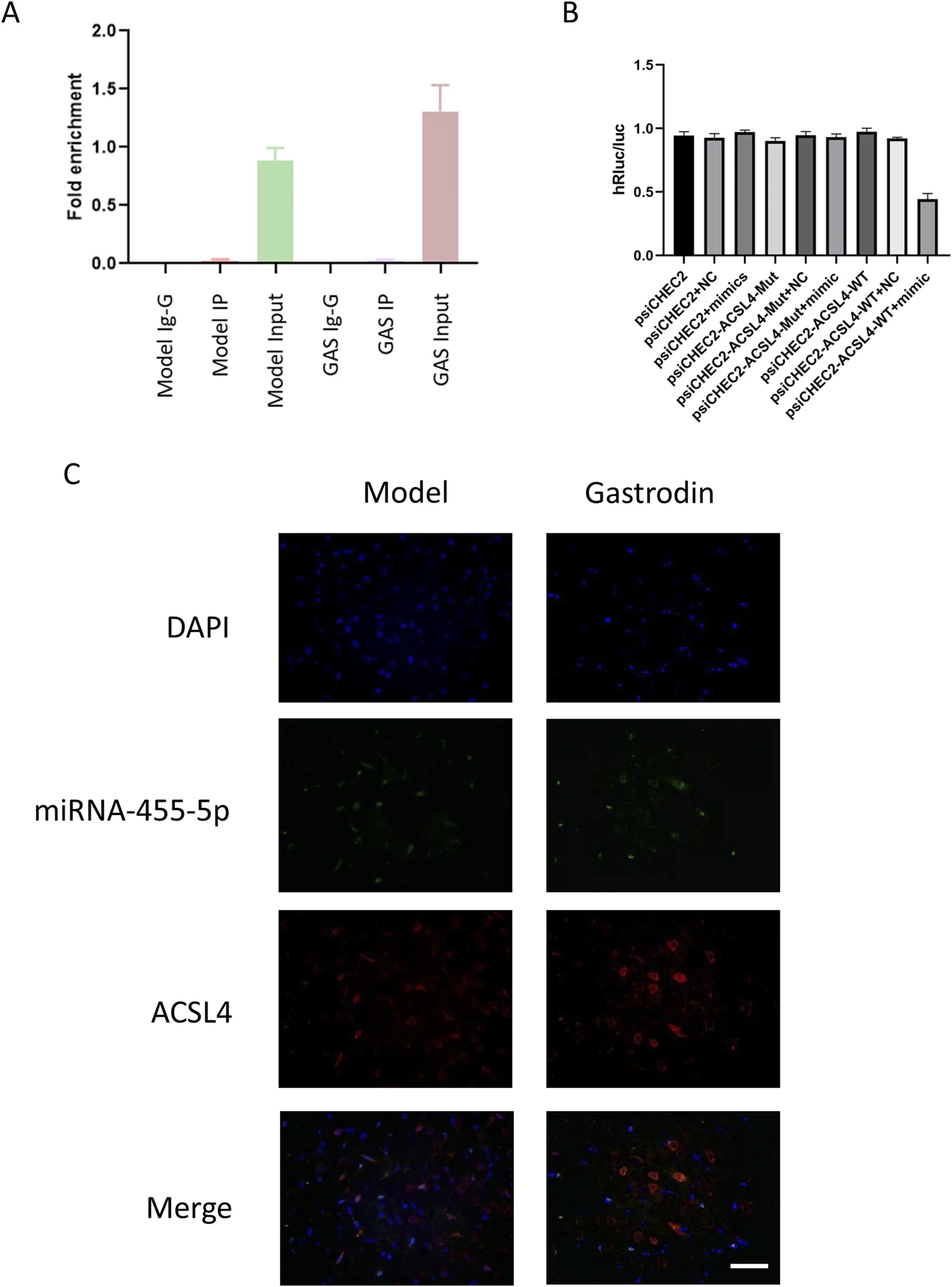
Gastrodin-treated Serum Exosome (GSE) can inhibit ferroptosis by regulating miR-455-5p/ACSL4 pathway via exosome. **(A)** R-IP result showed miR-455-5p does not directly interact with ACSL4 protein in MCAO rats. **(B)** Dual-luciferase reporter assay verified miR-455-5p directly targets the 3′-UTR of ACSL4 mRNA in Neuro-2a. **(C)** Compared with the model group, gastrodin treatment increased the fluorescence intensity and enhanced the co-localization signal of miR-455-5p and ACSL4 mRNA. Scale bar 20μm.

Furthermore, FISH analysis was performed in Neuro-2a cells to verify the subcellular co-localization between miR-455–5p and ACSL4 mRNA. Compared with the model group, gastrodin treatment increased the fluorescence intensity and enhanced the co-localization signal of miR-455–5p and ACSL4 mRNA, suggesting an increased spatial association between miR-455–5p and ACSL4 mRNA after gastrodin treatment (Figure 5C).

After that, we upregulated and downregulated the transcription level of miR-455–5p in Neuro-2a cell, and detected cell viability of each group by CCK-8 assay and live/dead assay. The results showed that GSE could rescue cell viability after miR-455–5p up/downregulation, indicating an association between the protective effect and miR-455-5p-related signalling pathway Figures 6A,B.

**FIGURE 6:**
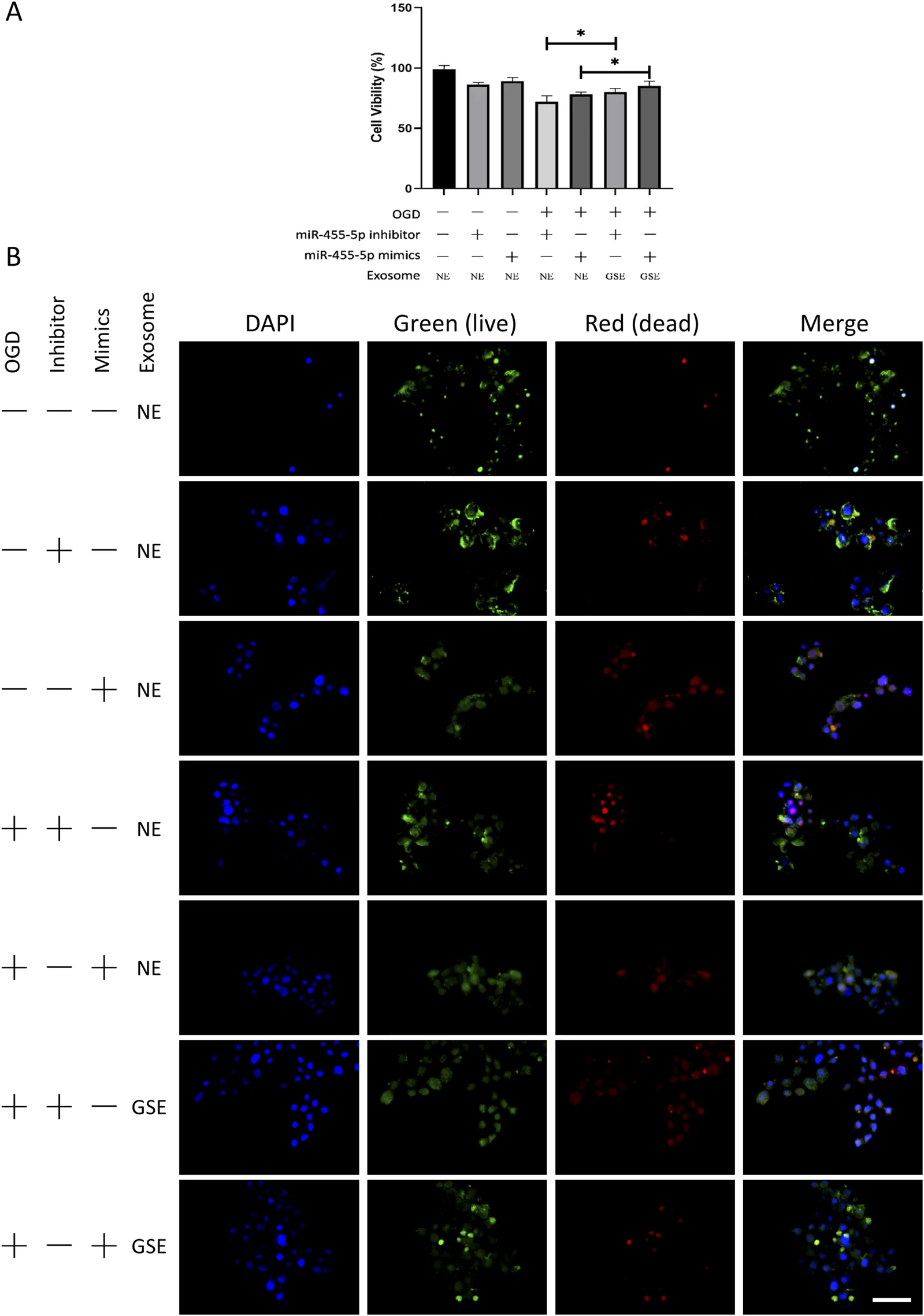
Gastrodin-treated Serum Exosome (GSE) can increase cell viability via miR-455-5p-related signalling pathway. **(A)** CCK-8 result showed that GSE could rescue cell viability after miR-455-5p up/down-regulation in Neuro-2a (n = 3). **(B)** Live/dead assay result showed that GSE could rescue cell viability after miR-455-5p up/down-regulation in Neuro-2a (Inhibitor represents miR-455-5p inhibitor, Mimics represents miR-455-5p mimics) (n = 3). *P < 0.05, **P < 0.01. Scale bar 20μm.

## 4 Discussion

As a natural bioactive compound, gastrodin exhibits multi-target protective effects in various ischemic and neurodegenerative diseases. In ischemic stroke models, gastrodin attenuates neuronal injury by, on one hand, suppressing the mtDNA/TLR9 and JAK2/STAT3 signaling pathways to interrupt the crosstalk between oxidative stress and inflammation (Zhang et al., 2024), and on the other hand, modulating the xCT/GPX4 and ACSL4/LPCAT3 pathways to effectively inhibit ferroptosis (Gong et al., 2025). In Alzheimer’s disease models, gastrodin mitigates microglial overactivation and neuroinflammation through regulation of the TLR4/TRAF6/NF-κB pathway (Wang et al., 2024). Furthermore, in myocardial ischemia-reperfusion injury, gastrodin enhances mitophagy via activation of the PINK1/Parkin pathway, thereby conferring perioperative cardioprotection (Chen et al., 2024). Collectively, these findings suggest that gastrodin may exert broad-spectrum protective effects against ischemic injury and neurodegenerative pathologies in distinct organs such as the brain and heart, through cross-regulation of key signaling cascades involved in inflammation, oxidative stress, ferroptosis, and mitochondrial quality control. The underlying mechanisms share common features (e.g., TLR-related inflammatory pathways) yet display tissue-specific characteristics, providing experimental evidence for the development of multi-target therapeutic strategies based on gastrodin. Our research offers a new perspective from the aspect of exosomes.

The present study provides novel evidence that gastrodin exerts neuroprotective effects against ischemic brain injury by suppressing ferroptosis through the exosome-mediated miR-455–5p/ACSL4 signaling pathway. This mechanism not only contributes to the growing understanding of ferroptosis in stroke pathology but also introduces an innovative approach to overcoming the blood–brain barrier (BBB) limitation via exosomal delivery.

Our findings confirm that gastrodin significantly reduces cerebral infarct volume in MCAO rats and restores neurological function, consistent with prior studies demonstrating its antioxidative, anti-inflammatory, and anti-apoptotic effects in ischemic models (Nan et al., 2023). Gong et al. recently showed that gastrodin modulates ferroptosis via the xCT/GPX4 and ACSL4/LPCAT3 pathways (Gong et al., 2025). Similarly, our study observed decreased ACSL4, TFR1, and MBL2 expression and increased GPX4 levels, suggesting that gastrodin protects neurons by attenuating ferroptotic processes. Ferroptosis, a regulated form of cell death driven by iron-dependent lipid peroxidation, has been recognized as a major contributor to neuronal loss in ischemic stroke (Dixon et al., 2012; Jiang et al., 2021; Long et al., 2025).

An increasing number of studies have focused on the critical role played by ACSL4 in the process of cerebral infarction. Recent neurovascular research identifies ACSL4-mediated ferroptosis as a primary driver of neuronal death and neuroinflammation following cerebral infarction (Cui et al., 2021). Ischemia-reperfusion injury triggers intracellular iron overload and reactive oxygen species (ROS) generation. Subsequent ACSL4 activation amplifies lipid peroxidation, expanding the infarct penumbra and worsening neurological deficits (Long et al., 2025). Pharmacological blockade of ACSL4 effectively suppresses lipid peroxidation, rescues GPX4 activity, and reduces brain infarct volume in vivo stroke models (Chen et al., 2021). Emerging studies reveal that complex upstream networks, such as the circ_0008146/miR-342–5p/ACSL4 non-coding RNA axis and HIF-1α, modulate ACSL4 expression during ischemic stress. These represent novel biomarkers and therapeutic targets for acute ischemic stroke (Liu et al., 2024).

GPX4 is a pivotal enzyme that detoxifies lipid hydroperoxides, maintaining redox homeostasis (Yang et al., 2014). Depletion of GPX4 leads to accumulation of lipid peroxides, resulting in ferroptotic death (Conrad and Friedmann Angeli, 2015). Our findings that gastrodin and GSE upregulate GPX4 expression indicate that this pathway is a central mediator of their protective effect.

Moreover, the downregulation of ACSL4 and TFR1 observed in this study aligns with evidence that these proteins enhance susceptibility to ferroptosis by promoting lipid peroxidation and iron uptake, respectively (Doll et al., 2017; G et al., 2015). The inhibition of these pro-ferroptotic regulators by gastrodin further supports its therapeutic potential in ischemic injury.

A major innovation of this work is the construction of gastrodin-treated serum exosomes (GSE). Exosomes, nanosized extracellular vesicles capable of crossing the BBB, have recently emerged as effective drug delivery vehicles for central nervous system diseases (Alvarez-Erviti et al., 2011). Previous studies have shown that exosome-mediated delivery enhances the stability, targeting, and bioavailability of therapeutic molecules (Tenchov et al., 2022). Our results demonstrate that GSE enhances cell viability and inhibits ferroptosis in Neuro-2a cells under OGD conditions, confirming the efficacy of this delivery strategy.

Mechanistically, our data identify the miR-455–5p/ACSL4 axis as a crucial mediator of GSE’s anti-ferroptotic effect. MicroRNAs have been increasingly recognized as post-transcriptional regulators of ferroptosis in neurological disorders (Mahmoudi-Lamouki et al., 2023). For instance, miR-137 and miR-9 have been reported to modulate ferroptotic death by targeting SLC7A11 and ACSL4 (Candia Plata et al., 2025; Do et al., 2025). We demonstrated that miR-455–5p directly interacts with ACSL4 mRNA, as validated through dual-luciferase and FISH analyses. Similar to our results, Zhang et al. reported that miR-455–5p attenuates oxidative stress-induced neuronal injury by activating the Nrf2 pathway, suggesting its broader neuroprotective role (Zhang et al., 2022).

Furthermore, the regulatory role of the miR-455–5p/ACSL4 axis links ferroptosis inhibition to oxidative stress mitigation and mitochondrial preservation, which are vital for neuronal survival (Cui et al., 2021). The upregulation of GPX4 observed here likely enhances glutathione metabolism and lipid peroxide clearance, thereby reducing oxidative burden and maintaining neuronal integrity (Hambright et al., 2017).

Collectively, our findings delineate a multifaceted neuroprotective mechanism in which gastrodin suppresses ferroptosis through the miR-455–5p/ACSL4 pathway probably via exosome. This strategy may overcome pharmacokinetic limitations and enhance therapeutic outcomes in ischemic stroke.

However, our research still has many shortcomings. GSE does not refer to gastrodin-loaded exosomes, it refers to gastrodin-stimulated exosomes. The constituent in GSE remains to be explored.

We acknowledge several limitations regarding the specificity of the observed EV-mediated protection. While our data demonstrate that GSE contains significantly higher levels of miR-455–5p than NE and that this miRNA is functionally required for neuroprotection, we have not conclusively excluded the possibility that co-isolated soluble factors—such as residual free gastrodin, serum proteins, lipoproteins, or other non-vesicular components—may contribute to the observed effects. Future studies with larger sample volumes and additional control groups (EV-depleted supernatant, detergent-disrupted vesicles, RNase protection, and free drug controls) will be required to definitively establish the exclusivity of GSE-mediated effects.

Also, further investigations are warranted to elucidate the *in vivo* biodistribution of GSE, confirm long-term safety, and evaluate its efficacy in large animal models or clinical settings.

## Data Availability

The original contributions presented in the study are included in the article/Supplementary Material, further inquiries can be directed to the corresponding authors.
